# Unveiling the threat of crystalline silica on the cardiovascular system. A comprehensive review of the current knowledge

**DOI:** 10.3389/fcvm.2025.1506846

**Published:** 2025-02-14

**Authors:** Irina Luciana Gurzu, Claudia Mariana Handra, Isabel Ghita, Marina Ruxandra Otelea

**Affiliations:** ^1^ Preventive and Interdisciplinarity Medicine Department, “Grigore T Popa” University of Medicine and Pharmacy, Iasi, Romania; ^2^ Occupational Medicine Department, “Carol Davila” University of Medicine and Pharmacy, Bucharest, Romania; ^3^ Pharmacy and Pharmacology Department, “Carol Davila” University of Medicine and Pharmacy, Bucharest, Romania

**Keywords:** silica exposure, silicosis, cardiovascular diseases, cardiovascular monitoring, biomarkers

## Abstract

**Introduction:**

This paper aims to expose the link between occupational exposure to respirable crystalline silica (SiO2) and cardiovascular diseases (CVDs).

**Methods:**

A comprehensive review of the literature was conducted, focusing on epidemiological studies that assessed the association between silicosis or SiO2 exposure and CVDs. Specific cardiovascular diseases, such as acute myocardial infarction, arrhythmias, pulmonary hypertension and pericarditis, were also pointed. Biomarkers commonly used in both silicosis and cardiovascular diseases were reviewed to underline the common pathological pathways.

**Results:**

Published epidemiological data revealed a higher risk of ischemic heart disease, stroke, and hypertension in silica-exposed workers, even at low exposure levels. SiO2 exposure was linked to an increased risk of myocardial infarction, with potential mechanisms involving inflammation and platelet activation. Elevated risk of arrhythmias, particularly atrial fibrillation, correlated with occupational silica exposure. Consistent with the pathological mechanisms supporting the SiO2 exposure—CVDs relationship, biomarkers related to NLP3 inflammasome activation, reflecting oxidative stress, and revealing fibrosis have been presented.

**Conclusion:**

Actual data support the relationship between occupational SiO2 exposure and various CVDs promoting cardiovascular monitoring in silica-exposed workers. Further studies are needed to identify specific/distinctive biomarkers to improve early detection of CVDs in silica exposed workers.

## 1 Introduction

Although known for centuries, silica exposure continues to generate occupational diseases all around the globe. In many historical workplaces with high exposure levels (mines, foundries, etc), the incidence has gradually decreased because of better protective measures, but in newly described occupations at risk, such as the stone benchtop industry, the incidence reached as much as 39.6% of workers (1).

Respirable crystalline silica (SiO2) is a well characterized risk factor for the lung. In addition to silicosis, SiO2 is carcinogenic for the lung (2). More recently, mutagenic effects on cultured cells have also been described for amorphous forms (3). Connective tissue disease was reported in 11.4% of the patients with silicosis (4). They are not necessarily related to the previous century's high levels of exposure, as sporadic cases of association with autoimmune diseases, such as rheumatoid arthritis and scleroderma have been reported in recent years (5, 6). In the Danish working population followed from 1997 to 2015, the risk of idiopathic interstitial pneumonias and pulmonary sarcoidosis was also directly related to cumulative exposure to SiO2 (7).

Apart from lung diseases, exposure to SiO2 is associated with a 40% higher risk of chronic kidney disease (8). A certain impairment of renal function might be found in 33% of patients with silicosis (9).

With increased awareness of the effects of inhaled microparticles on the cardiovascular system, the correlation between exposure to SiO2 and cardiovascular diseases has also been highlighted in recent publications. This relationship goes beyond the expected pulmonary heart disease secondary to lung fibrosis and, at least in some analyses, is present even at low exposure levels. Because cardiovascular disease is the number one cause of mortality in many countries worldwide, and the low level of exposure to SiO2 is not very rare, we have reviewed the most frequently documented cardiovascular diseases reported in the literature.

## 2 Methods

A comprehensive review of the literature was conducted, focusing on epidemiological studies that assessed the association between silicosis or SiO_2_ exposure and CVDs. The approach involved three distinct steps. In the first step, we searched for epidemiological data on the association between silicosis (or SiO2 exposure) and the incidence or mortality of cardiovascular diseases, exploring existing database such as Web of Science and Medline. The inclusion criteria were full-text, open-access articles published in English, before 2024, containing in their titles/abstract the keywords (“silicosis OR silica exposure”) AND (“cardiac OR vascular disease”). The sole exclusion criteria was duplication of articles across the searched databases. Based on this initial overview, in the second step, We searched for a more in-depth description of distinct cardiovascular diseases (acute myocardial infarction, arrhythmias, diseases of the pericardium, pulmonary hypertension, and cardiovascular impairment in autoimmune diseases related to silicosis) to better comprehend the impact of silica on the cardiovascular system. For each disease the search terms were “silicosis OR silica exposure AND the designated cardiovascular disease. Finally, we described common serum biomarkers for silicosis and the cardiovascular diseases to explain the epidemiological findings and extend the understanding in this area. For this section of the review, we searched in the Web of Science database (key words: “silicosis” AND “biomarkers”) and excluded the articles looking for biomarkers in the exhaled breath or broncho-alveolar lavage, for the genetic or epigenetic markers, or silica-related cancer. Searches that were confirmed in at least two independent studies were verified for being considered in the early detection or prognosis of the cardiovascular disease.

We aimed to select articles that explicitly intended to cover both domains (SiO_2_ exposure/or silicosis and cardiovascular diseases). We have prioritized studies with large number of participants (at least tens of workers), multicentric or representative at the national level, and whenever possible, those combining clinical with the pathological point of view and examining data from multiple perspectives.

## 3 Cardiovascular diseases associated with exposure to respirable crystalline silica

### 3.1 The overall association between silicosis or SiO2 exposure and cardiovascular diseases

While an association with pulmonary heart disease might be anticipated, as silicosis is a chronic pulmonary fibrosis, a surprisingly higher risk for ischemic heart disease was also found in workers with exposure below or at the permissible exposure limit [hazard ratio [HR] = 1.09, 95% [confidence interval] CI: 1.02–1.16] (10). Similar results were found in a cohort of 74,000 workers exposed to SiO2, in which mortality from ischemic heart disease was increased only in those with low exposure to non-combustion-sourced particles of crystalline silica (11). In a Swedish cohort study, the standardized incidence rate (SIR) of hospitalizations among male workers from iron foundries was significantly higher for ischemic heart diseases (SIR = 1.17, 95% CI = 1.07–1.29) and cerebrovascular diseases (SIR = 1.23, CI = 1.08–1.39) (12). Interestingly, as in the previous reports mentioned above, only the lowest quartiles of cumulative exposure had a statistically significant SIR with ischemic heart disease. A particular finding in this cohort was a high standardized mortality rate (SMR) from stroke (SMR = 1.61, 95% CI = 1.18–2.14) (13); particularly, data on smoking, and other occupational hazards (e.g., shift work, noise, carbon monoxide) were not included in the analysis.

If only non-smokers were considered, the association became relevant for ischemic heart disease and hypertension. In a study covering 16,918 non-smokers, the estimated SMR for ischemic heart disease was 1.18 (95% CI = 1.01–1.37) and hypertension 2.23 (95% CI = 1.86–2.65), respectively. Furthermore, these figures were even higher for workers with lower or medium levels of exposure (14). In this investigation, the SMR for cerebrovascular disease in non-smokers was lower than expected (SMR = 0.86, 95% CI = 0.79–0.93).

Finally, the meta-relative risk for ischemic heart disease estimated in a systematic review of SiO2 exposure was marginally significant (meta-relative risk = 1.07, 95% CI =1.00–1.16, *p* = 0.058). Remarkably, the risk was nonlinear in relation to the cumulative exposure, with most studies reporting the risk even at low levels (15).

### 3.2 Association of silicosis with different cardiovascular diseases

**Figure 1:**
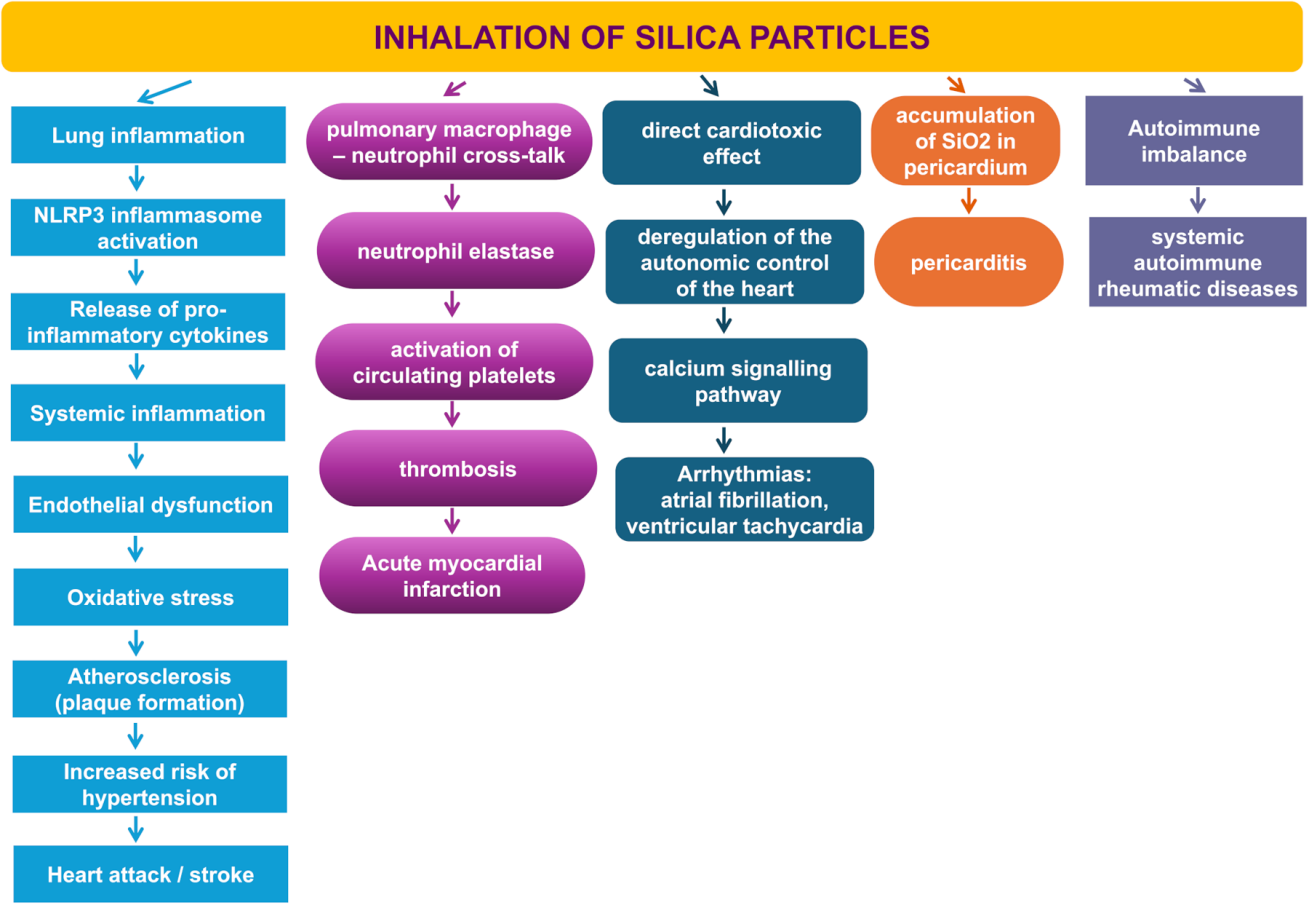
The association between silicosis or SiO2 exposure and cardiovascular diseases.

Silicosis and exposure to respirable crystalline silica (SiO2) are associated with particular cardiovascular diseases like acute myocardial infarction, arrhythmias, diseases of the pericardium, and cardiovascular impairment in autoimmune diseases related to silicosis (Figure 1).

#### 3.2.1 Silicosis and acute myocardial infarction

In search of the explanation for the highest mortality from cardiovascular diseases in silicosis, a comprehensive analysis of all manual Swedish workers on the association between SiO2 exposure and acute myocardial infarction (AMI) was performed. Data obtained from the national registries from 1992 to 2006 found an increased hazard risk, namely 1.66 (95% CI 1.27–2.18) in women and 1.06 (95% CI 1.03–1.10) in men, respectively. The risk of AMI was correlated with the cumulative exposure (16). Similar results were obtained in a uranium miners study, in which the incidence of AMI remained significantly higher, even after adjustment for smoking and metabolic syndrome (17). This case control study compared workers by quartile of exposure with controls never exposed occupationally to SiO2. Miners who had a long exposure time (31 years, on average), started their employment at a younger age (19 years, on average), and had a cumulative exposure either in the median or in high tertile had significantly higher odds ratios of AMI. A possible explanation for these results came from an experimental study, which showed that, in contact with SiO2, the pulmonary macrophage–neutrophil cross-talk releases neutrophil elastase into the blood circulation, which triggers the activation of circulating platelets (18). Concordant with this *in vitro* experiment, is the unusual thrombophilic response during the standard transplant surgery procedures described in a patient with silicosis. This patient developed a massive hollow catheter thrombosis from the right external iliac vein to the inferior vena cava (19), non-responsive to heparin.

#### 3.2.2 Silicosis and arrhythmias

Montén et al. investigated 5,508 men working in an occupation with possible exposure to quartz in the last five years prior to the diagnosis of atrial fibrillation (AF) diagnosis (20). The authors highlighted an increased risk of developing atrial fibrillation in male subjects aged between 20 and 55 years with an average exposure to quartz dust above 0.05 mg/m3. When the analysis was performed according to the number of years of exposure, the only statistically significant relationship was with the those with less than one year of exposure, which might be due to the healthy worker effect. The authors claimed that the rapid onset of arrhythmia by extrapolating data from environmental or experimental studies on ultrafine particles. These studies demonstrated a direct cardiotoxic effect on cardiomyocytes, deregulation of the autonomic control of the heart by reflexes initiated from lung inflammation, or initiation of systemic inflammation by the particles absorbed in the systemic circulation (21–23). It has also been proven that silica nanoparticles down-regulate genes involved in the calcium signalling pathway of the cardiac muscle (24).

There are also arguments in a case report of a patient with silicosis who developed monomorphic ventricular tachycardia (25). The patient had normal echocardiogram and normal coronary arteries confirmed by angiography. The cardiac MRI showed normal ventricular function but suggested left ventricular basal and mid-cavity wall striae of fibrosis, which was confirmed by endomyocardial ventricular septum biopsy. The authors suggested that SiO2 reduces sarcoplasmic reticulum Ca-ATPase activity, inducing dysfunction of cytosolic calcium dynamics and even apoptosis, as described in experiments with silica nanoparticles (26). Their hypothesis was supported by the observation that ventricular tachycardia did not respond to amiodarone and overdrive pacing but showed a positive response to phenytoin.

#### 3.2.3 Silicosis and pulmonary hypertension

The relation between silica exposure/silicosis and pulmonary hypertension (PH) is quite complex and not all silicosis patients would fit in the same clinical classification of this disease, as stated in the 7th World Symposium on Pulmonary Hypertension (27).

SiO_2_ particles accumulated in the pulmonary interstitium induce the local chronic inflammatory response with macrophage pyroptosis, which progresses towards the formation of granulomatous inflammation and pulmonary fibrosis (28, 29). The alteration of pulmonary architecture generates small airway dysfunction, leading to ventilation-perfusion mismatch and hypoxia, generating a group 3 PH. This would correspond, in general to patients with chronic simple silicosis. According to the new definition of HP (pressure in the pulmonary artery >20 mmHg), 59.6% of patients with chronic simple silicosis would have HP, most frequent in former perforators with probably higher exposure levels (30).

There are also pathological modifications supporting a group one PH, the arterial pulmonary hypertension (PAH). From the pathological point of view, the direct effect on the pulmonary artery could be classified in: (a) direct vessel injury and (b) the extrinsic, mechanical compression, although a combination of these mechanisms might be present in the same patient.

The direct vessel injury is supported by experimental data on animal models which showed that, in silica exposure of mice unprotected by superoxide dismutase to oxidative stress, the pulmonary vascular remodelling is present both in silicotic nodules and in the unaffected lung. This large distribution of the vascular damage leads to PAH and right ventricle failure (31). Few clinical data (mainly from case reports) support this finding. For example, a bluestone worker with simple chronic silicosis who had normal diffusion capacity and no restrictive disease, presented with PH, that responded to tadalafil and diuretics treatment. Therefore, the considered diagnosis was PAH (32). Silica exposure was also mentioned in a case of pulmonary capillary hemangiomatosis (33), a rare disease included in the PAH with features of venous/capillary (PVOD/PCH) involvement category. The contribution of the occupational exposure remains uncertain in this case, as the exposure to silica was short (2 years) and there were no signs of acute or sub acute silicosis on CT or biopsy features to prove the silica burden. Even more, if the working conditions might have been the cause, the patient was also exposed to organic solvents, a hazard which, in a case control study, significantly increased the risk of PVOD (adjusted OR of 12.8, 95% CI 2.7–60.8) (34). Therefore, even than other possible risk factors have been excluded by the authors, there are no sufficient arguments for an occupational relation.

The extrinsic compression of the pulmonary arteries is better documented. The prevalence of PH in progressive massive fibrosis is quite high (22.8%–33.9%) mostly in the ones with large (group C silicosis) and central location of the fibrosis (35, 36). Case reports have described pulmonary artery stenosis because of the calcified hilar lymph nodes which distorted the normal tissue architecture (37) simultaneous fibrotic stenosis of the large branches of the pulmonary artery and pulmonary vein (38), or fibrosing mediastinitis (39). All these cases were treated by vascular stenting with significant improvement of the clinical status.

As to further complicate the clinical diagnosis, there is also the possibility of a myocardial fibrosis associated with silicosis (which, eventually lead to a PH group 2 (25).

As consequence, PH is a relatively frequent complication of silicosis and should be evaluated no matter the stage of silicosis. The only conclusion which can be raised from these sporadic cases is that there not a single mechanism explaining the PH, but that deciphering the pathogenic mechanism in a particular patient is every important, as different treatments might be indicated.

#### 3.2.4 Silicosis and pericarditis

The inhalation of respirable crystalline silica is followed by the deposition of SiO2 in lung tissues; however, the accumulation of SiO2 in the serous membranes is also possible (40).

Pericardial damage associated with silicosis may be due to SiO2-induced immune reactions or by the well-known association with tuberculosis. To the best of our knowledge there are no epidemiological studies on the prevalence of pericarditis in silicotic patients or in workers exposed to silica. The search of the main medical databases (Web of Science, PubMed, Science Direct) revealed several interesting and well documented cases of pericarditis in which a thorough examination excluded bacillary etiology. Mohebbi et al. reported a clinical case of a worker who died at the age of 19 years due to cardiogenic shock secondary to the cardiopulmonary complications of accelerated silicosis that developed over 18 months of work at a stone grinding factory. Echocardiography showed bi-ventricular hypertrophy, and autopsy confirmed cardiomegaly and described a pericardial plaque and pericardial effusion of 450 ml (41). In another case of silicosis in a former miner, the pericardial effusion was massive enough to require surgical treatment (pericardial fenestration), in which case, the biopsy revealed white nodules on the pericardial surface containing lymphocytic infiltrate and hyalinized fibrosis (42). There is also a report of constrictive pericarditis accompanying silicosis in a stone quarry worker, in which the biopsy showed granular and fibrous tissue hyperplasia (43). Although the translocation of inhaled respirable crystalline silica through pulmonary and lymphatic capillaries was documented in a case of pleural effusion (44), these cases do not provide arguments for a possible translocation to the pericardium, as none of them found silica in the pericardium biopsies.

A meta-analysis found that silicosis is among the immunosuppressive conditions that increase more than 4 times the risk of tuberculosis (45). Exposure to respirable crystalline silica inhibits CD8T lymphocytes, impacts phagocytic activity, and compromises the viability of macrophages and neutrophils, leading to an increased vulnerability to mycobacterial infections (46) and/or facilitating the activation of the latent infection. The majority of cases are restricted to the lungs and pleura, with at least one report of tuberculous pleuritis and pericarditis in a patient with silicosis who had no contact with other tuberculosis patients (47). This patient was a smoker (13 pack-years) and worked in a sandpaper (abrasive paper) factory for five years without using protective masks. The specificity of this case was the apparently exclusive extrapulmonary localisation, as all attempts to diagnose pulmonary tuberculosis (culture, direct staining from broncho-alveolar lavage, culture and GeneXpert MTB/RIF assay in sputum) failed to detect Mycobacterium tuberculosis. The arguments for the origin of tuberculosis were the high value of the Adenosine Deaminase test in pleural and pericardial fluid and the clinical evolution following the antituberculosis treatment.

#### 3.2.5 Cardiovascular diseases in silicosis associated with systemic autoimmune rheumatic diseases

The literature provides sufficient evidence for the association between silicosis, or even occupational exposure levels of SiO2 and systemic autoimmune rheumatic (SARD) diseases (48). This seems to be directly related to cumulative exposure. The risk depends on the immunological conditions. For example, the incidence rate ratio of systemic sclerosis was 1.62 (1.08–2.44) in the exposed group compared with to the non-exposed group (49). For rheumatoid arthritis, the odds ratios were 1.94 (95% CI 1.46–2.58) in occupational exposure to SiO2 and this risk was even higher in seropositive smokers (50).

The presence of SARD increases the global cardiovascular risk. If one disease was present, the hazard ratio (HR) was 1.41 (95% CI 1.37–1.45). The risk almost doubles for two concomitant diseases and becomes 3.79 (3.36–4.27) if three or more SARDs are present (51). Premature atherosclerosis, endothelial dysfunction, microvascular damage, thrombosis, antiphospholipid syndrome, valvular heart disease, arterial stiffness, pericarditis, and hypertension have been described at various frequencies depending on the specific SARD (52–55).

Immunological dysfunction is assumed to contribute significantly to the high incidence of the cardiovascular diseases, and an indirect estimation of this dysfunction is the presence of the autoantibodies which characterize each distinct form of SARD. A meta-analysis estimated an incidence of venous thromboembolism of 12.4% in ANCA-associated vasculitis (56). Another meta-analysis calculated the relative risk for ischemic heart disease of 1.60 (95% CI: 1.39, 1.84) and cerebrovascular accidents of 1.20 (95% CI: 0.98, 1.48) (57). A 10-year longitudinal study of 2,803 participants, without cardiovascular and immunological disease at baseline, measured the antinuclear antibodies (ANA) at enrolment. Plasma ANA levels were higher in participants who developed hypertension (*p* = 0.02). The HR of mortality from cardiovascular disease adjusted for the classical risk factors (hypertension, diabetes, smoking, body mass index, estimated glomerular filtration rate, statin use, total cholesterol, triglycerides, and high-density lipoprotein cholesterol) was significantly higher: 1.37 (1.10–1.73). The HR was correlated with the ANA values (58). People with positive rheumatoid factor (RA), even without any joint symptoms, followed for at least 20 years, had a significantly higher mortality rate from cardiovascular diseases, after adjustment for other risk factors (HR 1.60, 95% CI 1.08–2.37) (59).

More than a fifth of workers have an autoimmune disease (60) in a multicountry study on patients with silicosis. A comparison between 203 patients with simple silicosis, 286 patients with complicated silicosis and 95 exposed workers without silicosis provide more specific results on the types of registered autoantibodies. In the total sample, ANA were found in 15.92% of the participants, antibody to extractable nuclear antigen (anti-ENA) in 9.24%, anti-neutrophil cytoplasmic antibodies (ANCA) in 4.5%, anti-neutrophil cytoplasmic antibodies (anti-CCP) in 0.02%, and RA in 16.78%. No statistically significant differences in positive autoimmune antibodies were found among the three groups, except for the ANA, which were less frequent in the exposed subjects (48).

Overall, the data suggest a triggering effect of SiO2 on the formation of the autoantibodies, even before the classical silicosis features are identifiable with our current diagnosis methods and in the absence of a well-defined SARD.

## 4 Biomarkers and possible common pathological pathways

Theoretically, silicosis is a preventable disease, but, like many other occupational diseases, the practice challenges this affirmation. Therefore, early detection plays a key role in the discontinuation of exposure and treatment of comorbidities.

For decades, researchers have tried to identify biomarkers that are easy to measure (in blood or urine), sensitive, and specific enough to replace chest radiography and reduce radiation exposure. For occupational diseases, early detection is as important as disease progression, so, there is generally a relationship between the duration and intensity of exposure and the severity of the disease.

**Figure 2:**
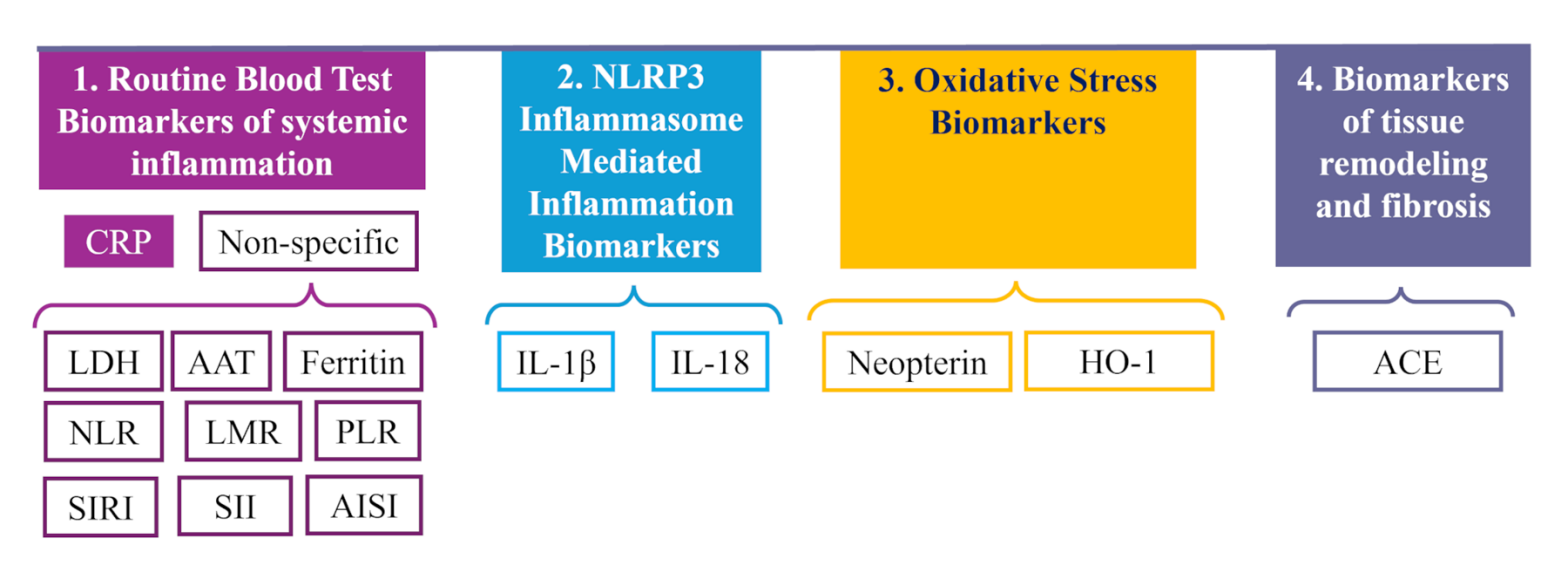
Biomarkers considered in the early detection or prognosis of the cardiovascular diseases in silicosis. CRP, C-reactive protein; LDH, lactate dehydrogenase; ferritin; ATT, alpha-1 antitrypsin; NLR, neutrophil-to-lymphocyte ratio; LMR, lymphocyte-to-monocyte ratio; PLR, platelet-to-lymphocyte ratio; SIRI, systemic inflammation response index; SII, systemic immune-inflammation index; AISI, aggregate index of systemic inflammation; IL-1, interleukin-1; IL-18, interleukin-18; HO-1, heme oxygenase-1; ACE, angiotensin-converting enzyme.

We synthesized the main common biomarkers to silicosis and cardiovascular diseases in: biomarkers from routine blood tests, biomarkers related to NLP3 inflammasome activation, biomarkers reflecting oxidative stress and biomarkers of fibrosis (Figure 2). The scope of this paper is not a systematic review of biomarkers proposed in the different stages of silicosis, but to illustrate the main pathological mechanisms of silicosis with the most frequently explored biomarkers that have also been considered in cardiovascular diseases.

### 4.1 Biomarkers from routine blood tests

These biomarkers have in common some advantages, such as the availability, the cost-/effectiveness and the standardization of the methods. They also have a major disadvantage: they lack specificity.

Lactate dehydrogenase (LDH), ferritin and *α*1anti-tripsin are classical markers of acute inflammation. Several indices, reflecting the proportion of different cells in the circulation, such as the neutrophil-to-lymphocyte ratio (NLR), lymphocyte-to-monocyte ratio (LMR), platelet-to-lymphocyte ratio (PLR), systemic inflammation response index (SIRI), systemic immune-inflammation index (SII), and aggregate index of systemic inflammation (AISI), were reported to differentiate between exposed and silicosis patients or between stages of silicosis (61–64).

All of them have been proposed for quantifying the risk or for monitoring patients with cardiovascular diseases, but none have reached the consensus as the C-reactive protein. There are some data also about the possible value of measuring C-reactive protein in monitoring the workers exposed to SiO2 (64, 65), but they are still not enough to draw a conclusion, although the results of these two studies complement each other: while the first showed a positive relationship with the duration of the exposure, the second only found differences between silicosis and exposed persons.

### 4.2 Biomarkers related to NLRP3 mediated inflammation

Activation of the nod-like receptor pyrin domain-containing-3 (NLRP3) inflammasome in lung macrophages after SiO2 inhalation are key element in the pathogenesis of silicosis. Due to NLRP3 activation, lung macrophages release active IL-1β and IL-18 (66), maintaining an inflammatory milieu and enhancing the transition of epithelial to mesenchymal cells, which promotes fibrosis (67). However, either through the leakage of these mediators or through the passage of very small nanomolecules of SiO2 into the circulation, the NRPP3 inflammation pathway was also activated in the peripheral monocytes collected at the end of the shift from workers in modern foundries (68) with a level of exposure lower than the permissible exposure limit in Europe. On the other hand, the results from different studies remain controversial, with higher (69) or no significant differences (65) recorded between exposed/non-exposed or severity stages of silicosis.

IL-1β is also a cytokine of interest for cardiologists. A plethora of experimental studies showed cardiac effects of IL-1β such as impairment in contraction due to deficits in stimuli transmission or (70) or the inhibition of the energy production (71). IL-1β is up regulated in acute ischemia and plays a role in the dilatative remodelling of the heart after acute myocardial infarction (72). Regarding the vascular effects, the pro-inflammatory role of IL-1β is up regulated in the earlier stages of the atherosclerotic disease and is the key regulator of the inflammation in the arterial tissue (73).

Another consequence of the NLRP3 activation is an increase in active IL-18, a marker of the TH1 cytokine profile. It was significantly higher in patients with simple silicosis compared to healthy controls, but also between simple and complicated silicosis. The levels of IL-1 and IL-18, among other cytokines, remained elevated after months of interruption of exposure to artificial stone (74), although its clear role (pro- or anti-fibrotic) remains to be confirmed (75). The significance of this biomarker in cardiovascular diseases is controversial. Plasma IL-18 increases vascular oxidative stress and the expression of matrix-metalloproteinases (76) in the early stages of atherosclerosis (77). Serum levels tend to increase in patients with metabolic syndrome, hypertension (78) and acute coronary syndromes (79). In a nested case-control study of 5,561 men followed up to 16 years, the highest tertile of IL-18 had, after adjustment for age, smoking, blood pressure, total cholesterol, HDL-cholesterol, body mass index, history of diabetes, chronic heart disease, social class and physical activity an odds ratio of 1.48 (95% CI = 1.15, 1.90) for coronary heart disease (80). However, further adjustment for high-sensitivity protein C or pro-brain natriuretic peptide attenuated the statistical significance (81).

Both IL-1β and IL-18 are good candidates for monitoring, but the current data are not sufficient for a conclusion. Findings of high levels after recent exposure should also be a trigger for cardiovascular pathology. The interest of research in this area is even higher, as therapeutic solutions for NLRP3 induced inflammation are under evaluation (82).

### 4.3 Biomarkers of the oxidative stress

Experimental studies have shown that the oxidative mechanisms promoted by SiO2 in the lungs might be extended to other tissues. For example, the mitochondrial dysfunction and the oxidative injury are also present in the myocardium (83). Promising results about the oxidative status in silicosis were obtained from measuring the serum levels of malonaldehyde (84) or catalase (85), but for the time being, further, independent, confirmation is needed. Concerning oxidative status, a high number of publications have referred to neopterin and heme oxygenase 1.

#### 4.3.1 Neopterin

Exposed vs. controls had higher neopterin values (86, 87) correlated with the silica levels in urine (88), which makes this biomarker suitable for monitoring exposure. Other research groups reported higher values of serum neopterin in silicotics compared to non-exposed to SiO2 controls and between the progressive stages of silicosis (89, 90). The explanation of these findings is still missing because the experimentally induced silicosis gave conflicting results on interferon gamma (IFN*γ*), the best characterized activator of neopterin (91), with some studies showing an increased level of IFN*γ* (92) and others finding only the up regulation of IFNs type I with apparently no major impact on the evolution of silicosis (93) or even a dysfunctional signal of IFN*γ* in macrophages loaded with SiO2 (94). An interesting, but small of study in 27 exposed workers might explain these conflicting results (95). In this study, the level of IFN*γ* was higher in exposed vs. non-exposed groups and very low in the four cases of silicosis, suggesting a variation of the expression of this cytokine during the evolution of the disease.

Regarding cardiovascular diseases, neopterin was associated with a worse prognosis in heart failure of non-ischemic causes (96), heart failure (97) acute coronary events (98), and peripheral artery disease (99, 100). In chronic coronary disease, the serum levels of neopterin were higher than those in patients without ischemic disease. In human endothelial cells, neopterin down regulated the NF-κB and the oxidative status of the cells, contributing to the reduction of the oxidative status, and suppressed the proliferative effect of angiotensin II on the muscular cells of the aorta. This, *in vitro* and in experimental atherosclerosis, showed that neopterin had a protective effect in the development of atherosclerosis either in exogenous administration or endogenous up regulation (101).

Different factors influence the serum level of neopterin such as recent diet (102), gender (103) and comorbidities. Most probably, the evolution of the disease influences the serum levels, and finding the proper significance of neopterin for screening, diagnosis and prognosis still requires longitudinal studies to measure the dynamics of this biomarker.

#### 4.3.2 Heme oxygenase 1

In the lungs, SiO2 particles are taken up by the macrophages via phagolysosomes. In the phagolysosomes, the NADPH-oxidase 2 and possibly other molecules generate reactive oxygen species (ROS), which leak into the cytoplasm and promote apoptosis (104). The ultrafine particles seem to be the most aggressive (105). Heme oxygenase 1 (HO-1) was detected in and around the silicotic nodules (106, 107).

HO-1 is constitutively expressed in a few cells but is highly up regulated under oxidative stress in cells involved in the defence mechanism. HO-1 catabolizes heme to biliverdin, bilirubin and CO. The first two have direct antioxidative effects, while CO acts through the inhibition of NADPH oxidase or other enzymes related to the ROS production (108). Even more, HO-1 switches the macrophages towards the M2-like phenotype (109), modulating the inflammatory process, but promoting fibrosis.

One hypothesis is that, in the early stages of silicosis, HO-1 acts as a compensatory mechanism, but as the disease advances and the oxidative stress persists, a gradual depletion of HO-1 occurs. In support of this hypothesis, higher levels of HO-1 were observed in workers exposed to SiO2 in limestone crusher (110) and stone-craving factories (111) and mines compared to unexposed (112). In a longitudinal study, low serum HO- levels 1 predicted the severity of the lung function decline (113). Another argument for the protective effect of HO-1 was derived from a surprising result in a group of artificial stone workers. In this study, HO-1levels in never smokers were negatively correlated with the number of years of exposure and were significantly lower in those with extensive pulmonary fibrosis. The explanation provided by the authors was that smoking induces the expression of HO-1, which limits the inflammation and the decline of the lung function (114).

However, not all studies agree with this pattern of the HO-1 variation in silicosis. For example, in study conducted on limestone workers already mentioned, the level of HO-1 was correlated with the intensity of exposure but was higher in those already diagnosed with silicosis (110). Another study also found indirect signs of persistent up regulation of HO-1 in patients with silicosis long time after the cessation of the exposure (115).

HO-1 also has a protective effect on the cardiovascular system with regard to the components of metabolic syndrome. HO-1 reduces the oxidative stress generated by sustained hyperglycemia, the accumulation of lipids in the adipocytes, the formation of the foam cells, protects against the mitochondrial damage and promotes mitochondria biogenesis in the myocardium (116). HO-1 opposes to the formation of the foam cells by reducing the levels of ROS, MCP-1 and interleukin 6 and by decreasing the internalization of lipids in the macrophages from the blood vessel walls (117). In later stages of the atherosclerotic plaques, HO-1 contributes to the plaque stabilization (118). Polymorphisms in the HO-1 promoter region which lower the expression of HO-1, are associated with a higher risk of cardiovascular disease (119).

Some studies suggest the same compensatory mechanism as in silicosis, with AMI, a high intracardiac HO-1 in AMI (120) and low expression in chronic diseases (ischemic heart disease or peripheral arterial disease) with a negative impact on mid-term survival (121, 122).

To a certain extent, several medications extensively used in cardiovascular diseases (such as statins or nicorandil) achieve their beneficial effects by activating HO-1 (123, 124). These drugs provided some encouraging results in experimental silicosis. For example, nicorandil upregulated Nrf2 and HO-1, showing promising results in experimental silicosis (125), whether statins downregulate endothelial mesenchymal transformation and oxidative stress (126).

Finally, the efficiency of the proposed treatment in experimental silicosis was partially explained by the regulation of HO-1 production (127). The same treatment was also found to be efficient for cardiac protection (128).

### 4.4 Fibrotic mechanisms

#### 4.4.1 Angiotensin converting enzyme

Angiotensin converting enzyme (ACE) was found sequent higher in healthy controls, exposed workers, simple silicosis and complicated silicosis in the largest and more recent studies on this topic (61, 129, 130). In the lung, ACE is produced by endothelial cells and macrophages and converts angiotensin I to angiotensin II (Ang II), mostly known for its direct cardiovascular effects mediated by the AT1 receptor: increase in sympathetic tone, vasoconstriction, retention of sodium, release of aldosterone and anti-diuretic hormone. Besides these effects, activation of AT1 by Ang II has fibrogenic consequences through the induction of fibroblasts proliferation, promotion of myofibroblasts differentiation and collagen deposition (131, 132). This has been proven by the suppressive effect of angiotensin convertase 2 or captopril on the ACE/angiotensin II/AT1 axis, which reduced the epithelial-mesenchymal transition in experimental silicosis (133, 134). In one of these experiments, the expression of ACE and AT1 gradually increased as the disease developed (134).

The experimental and clinical data are consistent about the role of the ACE in cardiovascular disease. ACE is part of the complex renin-angiotensin-aldosterone system and the balance between its two main axes (the ACE/Ang II/AT1 and the ACE-2/Angiotensin 1–7/MasR) directs the pathological progress. If not balanced by the ACE-2, ACE has been related to the development of fibrosis and hypertrophy of the myocardium, and with a high local sympathetic activity (135, 136). In the peripheral arteries, ACE-dependent production of Ang II favors inflammation, promotes monocytes adherence and ROS formation, contributing to atherosclerosis, dysfunctional endothelia and hypertension (137, 138).

There are several common elements for all the studies on biomarkers: in workers exposed to SiO2 or in silicosis, none of the results were adjusted for the presence of the cardiovascular disease, although cardiovascular diseases are frequent in any population. This becomes a major obstacle in generalizing the findings, and future research should clarify this possible bias. Another significant barrier for the validation of any of these biomarkers is the changing levels during the evolution of silicosis or cardiovascular disease. If both diseases are present, each of them might be in at a different stage and therefore contribute differently to what is measured in the blood. Further research should consider these issues to clarify if there is a biomarker for the impact of SiO2 on the cardiovascular system.

## 5 Conclusion

There is evidence of a relationship between SiO2 exposure and various cardiovascular diseases. This pleads for cardiovascular monitoring of exposed workers and even for the characterization of a new work-related disease due to SiO2.

The search for biomarkers, either for early detection or for progression of silicosis should not ignore the influence of possible co-existing cardiovascular disease. By clarifying the association between SiO2 exposure/silicosis and cardiovascular risk/disease, future studies should serve to a better monitoring of workers in many industries in which this exposure continues to be present.
